# Mutations in ace2 gene modulate cytokine levels and alter immune responses in *Mycobacterium tuberculosis* and SARS-CoV-2 co-infection: a Cameroonian cohort

**DOI:** 10.3389/fimmu.2025.1533213

**Published:** 2025-03-24

**Authors:** Mary Ngongang Kameni, Eric Berenger Tchoupe, Severin Donald Kamdem, Nikhil Bhalla, Jean Paul Assam Assam, Arnaud Njuiget Tepa, Fuh Roger Neba, Ranjan Kumar Nanda, Anthony Afum-Adjei Awuah, John Humphrey Amuasi, Palmer Masumbe Netongo

**Affiliations:** ^1^ Molecular Diagnostics Research Group, Biotechnology Centre-University of Yaounde I (MDRG-BTC-UYI), Yaounde, Cameroon; ^2^ Department of Microbiology, University of Yaounde I, Yaounde, Cameroon; ^3^ Translational Health Group, International Centre for Genetic Engineering and Biotechnology, New Delhi, India; ^4^ Department of Clinical Biochemistry, Faculty of Medicine and Biomedical Science, University of Yaounde I, Yaounde, Cameroon; ^5^ Department of Pathology, School of Medicine, University of Utah, Salt Lake City, UT, United States; ^6^ Kumasi Centre for Collaborative Research in Tropical Medicine, Kwame Nkrumah University of Science and Technology (KNUST), Kumasi, Ghana; ^7^ Department of Infectious Diseases Epidemiology, Bernhard Nocht Institute for Tropical Medicine, Hamburg, Germany; ^8^ College of Health Sciences, Kwame Nkrumah University of Science and Technology (KNUST), Kumasi, Ghana; ^9^ Biology Program, School of Science, Navajo Technical University, Crownpoint, NM, United States; ^10^ Department of Biochemistry, University of Yaounde I, Yaounde, Cameroon

**Keywords:** *ace2*, *tmprss2*, SNPs, COVID - 19, tuberculosis, *Mycobacterium tuberculosis*, SARS-CoV-2, immune response

## Abstract

**Introduction:**

SARS-CoV-2 and *Mycobacterium tuberculosis* (Mtb) share similarities in their modes of transmission, pathophysiological symptoms, and clinical manifestations. An imbalance in the immune response characterised by elevated levels of some inflammatory cytokines caused by tuberculosis (TB) and COVID-19 may increase the risk of developing a severe disease-like condition. It has been reported that TB increases the expression levels of Ace2 (angiotensin converting enzyme 2) and Tmprss2 (transmembrane protease serine 2) proteins, which are essential for COVID-19 pathogenesis. Single nucleotide polymorphisms (SNPs) variants of *ace2* and *tmprss2* genes can impact virus and host-cell interactions and alter immune responses by modulating cytokine production. This may modify the susceptibility and/or severity in COVID-19-infected people. The role of SNPs in *ace2* and *tmprss2* in relation to Mtb and SARS-CoV-2 co-infection is relatively underexplored.

**Method:**

In this study, genotype frequency of 10 SNPs of *ace2* and 03 SNPs of *tmprss2* genes in a Cameroonian cohort consisting of COVID-19-positive (n = 31), TB-positive (n = 43), TB-COVID-19 co-infected (n = 21), and a control group (n = 24) were studied. The immune response was estimated by quantitating inflammatory cytokine levels alongside self-reported and clinically diagnosed symptoms. The relationship between specific genetic mutations in these ace2 gene SNPs and their impact on cytokine expression levels in Mtb and SARS-CoV-2 co-infected patients was investigated.

**Results:**

We identified wild-type, heterozygous, and double-mutant genotypes in seven SNPs (rs2285666, rs6632677, rs4646116, rs4646140, rs147311723, rs2074192 and rs4646142) in *ace2* gene, which showed significant variations in distribution across the study groups. Our most significant findings include the association of double mutant alleles (AA) of rs4646140 and rs2074192 in the *ace2* gene with decreased IL-6 and IL-2 expression levels respectively in TB-COVID-19 participants. Also, the double mutant alleles (AA) of rs4646116 were responsible for increased expression level of IL-2 in TB-COVID-19 patients. Additionally, elevated serum levels of AST, urea, and D-dimer, as well as increased plasma concentrations of IL-10, IFN-γ, and TNF-α, have been associated with co-infections involving Mtb and SARS-CoV-2.

**Conclusion:**

These biomarkers may reflect the complex interplay between the two pathogens and their impact on host immune responses and disease progression. This study highlights the critical role of genetic and immunological factors in shaping altered immune responses during co-infections involving Mtb and SARS-CoV-2. By elucidating these factors, the findings provide a foundation for a deeper understanding of host-pathogen interactions and their implications for disease progression and outcomes. Furthermore, this research has the potential to drive advancements in diagnostic approaches enabling more accurate detection and monitoring of co-infections.

## Introduction

Tuberculosis (TB) is a global public health concern and is endemic in low middle-income countries, primarily those in sub-Saharan Africa and Asia. According to the Global TB Report 2024 by the World Health Organization (WHO), 8.2 million cases and 1.25 million deaths were reported in 2023, placing TB again as the leading infectious disease killer in 2023 (1). In 2020, the COVID-19 pandemic overtook TB-related deaths and incidences, making it the most contagious disease with the highest mortality. During this period, 3,931,534 cases and 100,952 deaths due to COVID-19 were reported in sub-Saharan Africa (SSA) (2). COVID-19, first reported in Hubei Province in China in December 2019, is caused by Severe Acute Respiratory Disease Syndrome Coronavirus 2 (SARS-CoV-2) (3). SARS-CoV-2 and *Mycobacterium tuberculosis* (Mtb) are major infectious causes of death. They do not cause similar diseases; however, they show few similarities in their modes of transmission, symptoms and manifestations (4). They transmit through respiratory tract secretions via aerosol mode and cause lower respiratory tract infections, pneumonia and associated lung fibrosis. Both diseases share similar clinical symptoms, such as cough, fever, weakness, and dyspnea. An imbalance of immune response, including significantly decreased absolute counts of T-cells and increased pro-inflammatory cytokines levels, may influence the risk of developing TB and COVID-19 diseases, among others (4). The cytokine storm causes a significant biological response identified in patients with severe COVID-19 due to the over-activation of the inflammatory cascade in the tissues exposed to harmful stimuli like injury, toxic chemicals or pathogens (5).

Coronavirus enters the host’s cell by binding its spike protein to the angiotensin-converting enzyme 2 (Ace2) receptor at the surface of the host. The *ace2* gene is located on the long arm of human chromosome 17 and consists of 26 exons and 25 introns (17q23.3) with a mass of ∼92.5 kDa (6). Ace2 catalyses the conversion of angiotensin I into angiotensin 1-9, and angiotensin II into the vasodilator angiotensin 1-7 (7)). It possesses a catalytic and collectrin-like domain that spans the membrane and makes it a surface protein. The spike protein of SARS-CoV-2 binds to the catalytic domain of Ace2, leading to a conformational change, thereby causing the viral entry into the host cell (8). Transmembrane protease serine 2 (Tmprss2) is a protein encoded by the *tmprss2* gene on autosomal chromosome 21q22.3, which regulates cell signaling and modulates host response to infection (9). However, in SARS-CoV-2 infection, *tmprss2* primes the viral glycoprotein by cleaving the spike protein at the S1/S2 junction and the S2 site (10). Both *tmprss2* and *ace2*, expressed in the lung bronchial epithelial cells, are critical for viral entry of SARS-CoV-2 (11). Some studies have demonstrated that *tmprss2* activity is essential for viral spread and pathogenesis, enabling the entry of SARS-CoV-2 in *ace2*-expressing cells but not in cells without *ace2* (12, 13). Ziegler and colleagues have shown that Mtb infection increases *ace2* expression in the lung tissues through interferon stimulation (14). Single nucleotide polymorphisms (SNP), which represent a change in a single DNA building block, are one of the most common types of genetic variation amongst humans. It usually plays a role in altering disease severity and/or susceptibility by altering the structure and function of a gene which encodes a protein or by affecting the binding of enzymes that regulate cellular processes among several others. In the context of COVID-19, SNPs in *ace2* and *tmprss2* genes have been studied in several populations to elucidate the impact on the establishment of COVID-19 (6). Susceptibility to SARS-CoV-2 infection could be defined by age, gender, pre-existing comorbidities, genetic background, predisposing factors such as health status, immunological state of the host and lineage of the pathogen (15). The host *ace2* and *tmprss2* genes play a vital role in disease severity, viral replication, and inflammation, and variations at genetic levels, such as SNPs, may affect their function (16). Mutations in SNPs of *ace2* and/or *tmprss2* genes could influence immune response by modulating cytokine production and may contribute to immune imbalance in Mtb/SARS-CoV-2 co-infected patients. Limited literature is available on the effect of SNPs in *ace2* and *tmprss2* and their response to COVID-19 and TB association. In this study, 95 participants with either TB and/or COVID-19 alongside a control group of 24 participants who reported no history of either TB or COVID-19 were recruited from a Cameroonian cohort. Their biochemical and immunological parameters were analyzed, followed by genotyping to investigate the mutation patterns in the *ace2* and *tmprss2* genes. The findings shed light on specific mutations in these genes that may impact susceptibility, severity, or immune dynamics in the context of COVID-19 and TB co-infection.

## Material and methods followed

### Ethics statement, study participants recruitment and classification

A written formal consent was obtained from the Cameroon National Ethical Committee for Research in Human Health (N° 2020/07/1265/CE/CNERSH/SP) in Yaoundé for this study. This study was carried out in compliance with bio-ethical laws and data protection state and following good clinical practice. All study participants recruited presented signs and symptoms of a respiratory disease. These participants were enrolled at the Djoungolo District Hospital, Jamot Hospital, Ekoumdoum Baptist Hospital and Red Cross Hospital in Yaoundé during the COVID-19 pandemic from September 2020 to December 2023. This study was conducted in two phases: a prospective component spanning from June 2022 to December 2023 and a retrospective component covering the period from September 2020 to December 2022. The prospective study enrolled participants from Jamot Hospital, including individuals diagnosed with TB and a control group for comparative analysis. In the retrospective study, participants were selected from Djoungolo District Hospital, Ekoumdoum Baptist Hospital, and Red Cross Hospital, focusing on individuals who were either COVID-19 positive or co-infected with TB and COVID-19. Recruited participants were assessed for medical history, tobacco consumption and TB medication history. We assessed the following variables: i) socio-demographic factors such as age, sex, occupation, number of co-inhabitants, ii) COVID-19 and TB history, asthma history, HIV status, hypertension, diabetes mellitus, tobacco and alcohol consumption, iii) clinical characteristics such as cough, fever, headache, sore throat, asthenia, chest pain, loss of smell. The vitals were measured by clinicians at the time of patient enrolment using a thermometer for temperature and a sphygmomanometer for heart rate and blood pressure. Other symptoms viz, cough, sore throat, bloody sputum, rhinorrhoea, chest pain, myalgia, arthralgia, fatigue, loss of smell and headache were self-reported by participants and noted by clinicians at the time of enrolment. Demographic details and clinical characteristics are presented in **Table 1**. Every participant underwent clinical examination and laboratory assessment for COVID-19, TB and other infectious diseases endemic in the region, such as Influenza A, B, malaria, HIV and Hepatitis B. A group of symptomatic individuals who tested negative for COVID-19, TB, and the other infectious diseases mentioned above, who had been screened during the study, were enrolled as controls. Nasopharyngeal samples, which tested positive for only COVID-19 by RDT and real-time PCR, were considered COVID-19 positive; sputum samples that tested positive for only TB by microscopy and real-time PCR were included as TB-positive participants and those which tested positive for both infections only were considered as TB-COVID-19 co-infected. Exclusion criteria for the study groups were existing comorbidities such as malaria, Influenza A and B, HIV, Hepatitis and unwillingness to give signed informed consent. Written and signed informed consent was obtained from every recruit before the start of the study. Study protocol and consent forms were reviewed and approved by the Centre’s regional ethical committee in Yaoundé. The study population was later classified into COVID-19 positive, TB positive, TB-COVID-19 association, and controls.

**Table 1:** Socio-demographic and clinical characteristics of study participants by study group (control, COVID-19, tuberculosis (TB), and TB-COVID-19).

Characteristics	Control (24)	COV (31)	TB (43)	TBCOV (21)	p-values
Age (mean ± SD)	30.5 ± 6.7	37.3 ± 12.8	36.8 ± 17.2	37.7 ± 14.5	ns
Male n (%)	11 (45)	17 (55)	32 (74)	13 (62)	N/A
Female n (%)	13 (55)	14 (45)	11 (36)	8 (38)	N/A
Temperature (°C)	37.2 ± 0.7	37.4 ± 0.7	37.6 ± 0.6	37.3 ± 0.8	ns
Heart Rate bpm (mean ± SD)	88.5 ± 16.4	87.5 ± 10.6	86.6 ± 10.5	87.5 ± 8.7	ns
Respiratory Rate bm (mean ± SD)	18.9 ± 1.3	16.8 ± 2.8	17.3 ± 3.1	17.9 ± 1.2	p<0.05
Systolic blood pressure BP/mmHg (mean ± SD)	117.8 ± 10.9	122.9 ± 19.0	125.3 ± 20.1	118.5 ± 8.4	ns
Diastolic Blood Pressure BP/mmHg (mean ± SD)	76.9 ± 8.8	80.7 ± 14.7	77.8 ± 13.8	79.8 ± 10.2	ns
O2 saturation % (mean ± SD)	98.0 ± 1.3	96.6 ± 1.8	97.9 ± 1.1	95.7 ± 3.2	p<0.05
Fever n (%)	11 (45.5)	22 (70.9)	20 (46.6)	15 (71.4)	P=0.05
Cough n (%)	12 (50)	23 (74.2)	23 (53.5)	18 (85.7)	ns
Sore throat n (%)	5 (22.7)	9 (29)	10 (23.3)	9 (45)	ns
Bloody sputum n (%)	0 (0)	1 (3.2)	7 (16.3)	2 (10)	p<0.05
Rhinorrhoea n (%)	10 (41.7)	12 (38.7)	7 (16.3)	7 (35)	ns
Chest pain n (%)	5 (22.7)	9 (29)	11 (25.6)	12 (57.1)	ns
Myalgia n (%)	5 (22.7)	12 (38.7)	9 (20.9)	8 (40)	ns
Arthralgia n (%)	12 (50)	6 (19.4)	9 (20.9)	11 (52.3)	ns
Fatigue n (%)	12 (50)	13 (41.9)	9 (20.9)	17 (80.9)	p<0.05
Loss of smell n (%)	5 (20.8)	9 (29.0)	6 (13.9)	6 (30)	ns
Headache n (%)	11 (45.8)	19 (61.3)	7 (16.3)	16 (76.1)	p<0.05

SD, standard deviation; ns, non-significant; bpm, beats per minute; bm, breathes per minute; °C, degree Celsius; O2, oxygen; COV, COVID-19; TB, Tuberculosis; TBCOV, Tuberculosis-COVID-19 association; NA, Not applicable.

### Nasopharyngeal sample collection and processing

Nasopharyngeal samples were collected from the participants by inserting the swab provided about 2 - 2.5 cm into the nostrils. The HIGHTOP Antigen Rapid Test device that Qingdao Hightop Biotech Company manufactured was used according to the manufacturer’s instructions. The QIAamp viral RNA mini kit extracted coronavirus RNA from nasopharyngeal samples. A confirmatory COVID-19 diagnosis was later made using real-time PCR using the Logix Smart ABC (Cat #: ABC-K-001) test utilizing the patented Co-Primer technology (17) according to the manufacturer’s instructions. The Co-Primer triplex assay uses extracted viral RNA to detect Influenza A, B and SARS-CoV-2 (gene RdRp and E-gene) in upper respiratory tract samples and even saliva (Netongo et al., unpublished data).

### Sputum sample collection and processing

Sputum samples were collected using plastic cups with 40 mL capacity. After collection, sputum microscopy was carried out. A slide was prepared for each sample, fixed, and later stained following the Zeihl-Nelseen staining technique. A confirmatory real-time PCR using the SARAGENE™ *Mycobacterium tuberculosis* test COSARA Diagnostics Ltd India and Logix Smart Mtb Kit (Cat #: MTB-K-007)-Co-Diagnostics inc, USA was performed on extracted bacterial DNA according to manufacturer’s instruction. This test detects the presence or absence of IS6110 and MPB64 genes from *Mycobacterium tuberculosis*. The test kit includes an internal control to identify possible qPCR inhibition and verify the quality of sample extraction.

### Blood sample collection and storage

5 ml of whole blood was collected into commercially available anticoagulant-treated tubes (EDTA) and dry tubes. The blood was centrifuged 5000 r.p.m for 10 mins. Plasma was obtained from blood collected in EDTA tubes, while serum was obtained from dry tubes. Both were then aliquoted and stored at -80°C for future use.

### Evaluation of biochemical markers assays involved in TB and COVID-19 diseases

A set of serum biochemical markers aspartate aminotransferase (AST), alanine aminotransferase (ALT), Urea, creatinine (CREA), direct bilirubin (BIL-D), total bilirubin (BIL-T) and D- DIMER. AST, ALT, UREA and CREA were quantified using the PreciseMAX reagent kit according to the manufacturer’s instructions and results read on the semi-automatic biochemistry analyzer. Serum levels of bilirubin were determined by the photometric detection of the azo derivatives obtained by the serum reaction with the diazonium ion of sulfamic acid. D-dimer was measured in serum using the dry fluoro-immunoassay analyzer (WWHS Biotech. Inc Shenzhen, P.R China).

### Measurement of pro-inflammatory and anti-inflammatory cytokine levels involved in COVID-19 and TB

Serum cytokines (IL-6, IFN-γ, TNFα, IL-10, IL-2 and IL-1β) were assayed in serum using sandwich ELISA Origene kits (Origene Technologies, Inc, Rockville, MD 20850, US) according to manufacturer’s instruction.

### DNA extraction and single nucleotide polymorphism genotyping

Genomic DNA was harvested from the peripheral blood using the commercially available Quick-DNA™ Miniprep Kit (QIAamp DNA Blood Mini kit, Qiagen, Germany), according to the manufacturer’s instruction and its quality was verified in agarose gels stained with ethidium bromide nucleic acid gel stain (Thermo Fisher Scientific, C.A, USA). Then, the DNA concentration was measured using nanodrop (Thermo Fisher Scientific, C.A, USA), and purity was determined by calculating the A260/280 ratio. The extracted genomic DNA was stored at -80°C until used in the genotyping reaction. SNPs were analyzed by real-time polymerase chain allelic discrimination technology using TaqMan SNP genotyping assay kit (Thermo Fisher Scientific, Waltham, MA, United States) on a Co-Dx Box Magnetic Induction Cycler qualitative Time Polymerase Chain Reaction (qPCR) machine (Co-Diagnostics Inc USA, Cat # MIC001355). The ten variants analyzed for the Angiotensin-converting enzyme gene (ACE 2) were rs2285666, rs4240157, rs4646142, rs4646116, rs6632677, rs4646140, rs147311723, rs2074192, rs35803318, rs4646179 and while three genotype variants were determined for the Transmembrane serine protease 2 Polymorphisms (TMPRSS2) gene, namely rs12329760, rs75603675 and rs61735791. Specifically, genotype variants were determined using the TaqMan™ SNP Genotyping Master Mix kit from Thermo Fisher Scientific, C.A, USA (Cat #: 4381656) that reveals Ace2 rs4646179 A>G, rs147311723 G>A, rs4646142 G>A, rs2074192 C>T, rs35803318 C>T, rs4646140 C>T, rs6632677 G>C, rs4646116 T>C, rs2285666 C>A, rs4240157 C>G and Tmprss2 rs12329760 C>G, rs75603675 C>A, rs61735791 C>A mutations.

The reaction mix of each sample was composed of 5 µL of 2X TaqMan Genotyping Master Mix, 0.5 µL of TaqMan assay (20X), and 4.5 µL RNase-free water. The thermal cycling protocol is optimized at 95°C for 10 min for AmpliTaq Gold, UP Enzyme Activation, followed by denaturation step at 95°C for 15 s and annealing/extension at 60°C for 1 min for 40 cycles. The qPCR was performed on a Co-Diagnostics PCR instrument (Co-Diagnostic, INC, Salt Lake City, USA), and the results were analyzed using Co-Diagnostic genotyper software. This software was used to plot the findings of the allelic discrimination data as a scatter plot of Allele 1 (VIC^®^ dye) versus Allele 2 (FAM™ dye). Each well of the 96-well reaction plate was represented as an individual point on the plot.

### Data analysis and management

The data was anonymized before analysis with numerical variables. All comparisons of cytokines and biochemical biomarkers data were analyzed using Graph Pad Prism 8.0. Arithmetic means, medians and standard deviation were also determined using built-in MS Excel 2016 Home Edition commands. Test techniques include independent student t-test and Oneway ANOVA test, one for comparing 2-independent groups and the other for more than 2-independent groups. SNP frequencies were expressed as numbers (%) in each group. The chi-square test was used to determine p-values and the association of genotypes with one of the groups.

## Results

### Socio-demographic and clinical characteristics of study participants

**Figure 1:**
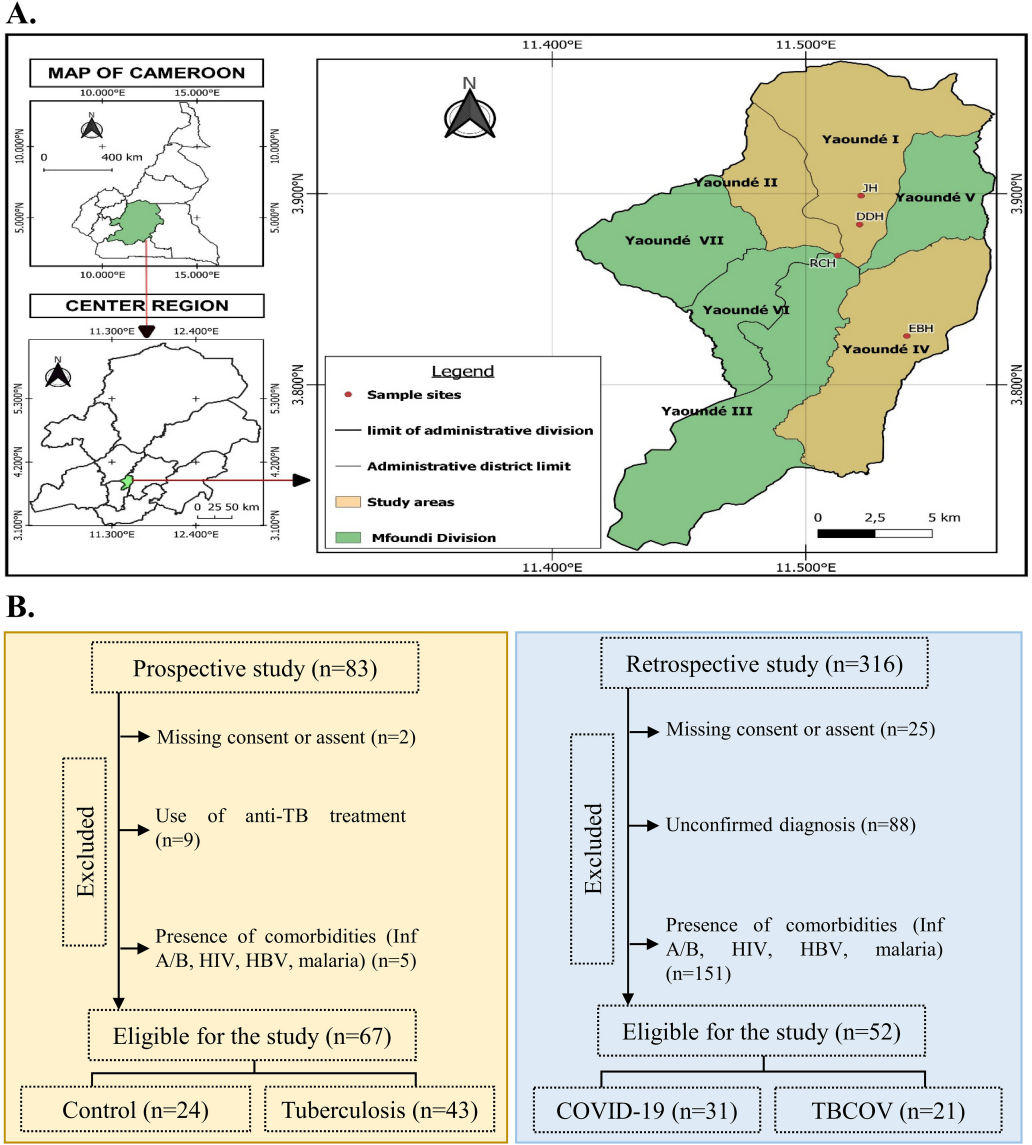
Study participant recruitment and classification. **(A)** Map showing the Centre region of Cameroon with different study sites. Samples for this study were collected at four hospitals: JH, DDH, RCH, and EBH, which are situated in Yaoundé. The sample sizes from each district were as follows: JH: 83; DDH: 102; RCH: 96; and EBH: 118. The map was created using QGIS version 3.32.3. **(B)** Based on COVID-19 and Tuberculosis infection status, participants were grouped into Controls: COVID-19 positive, Tuberculosis positive, and Tuberculosis and COVID-19 association. In total, our sample size consisted of 119 participants. JH, Jamot Hospital; DDH, Djoungolo District Hospital; RCH, Red Cross Hospital; EBH, Ekoumdoum Baptist Hospital.

All study participants presented signs and symptoms of a respiratory disease. Participants were enrolled at the Djoungolo District Hospital (102), Jamot Hospital (n=83), Ekoumdoum Baptist Hospital (n=118) and Red Cross Hospital (n=96) in Yaoundé during the COVID-19 pandemic from the period of September 2020 to December 2023. The location of the study site in the Centre region of Cameroon is illustrated in **Figure 1A**. Every participant underwent clinical examination and laboratory assessment for COVID-19 and TB. Recruited participants were assessed for medical history, tobacco consumption and TB medication history. From 399 participants, 280 were excluded from the study due to the presence of other comorbidities such as Influenza A and B, HIV, malaria and Hepatitis (n=156), use of anti-TB treatment (n=9), missing consent and unconfirmed diagnosis (n=115). In total, only 119 participants were retained for this study. The study populations consisted of four groups: COVID-19 positive (n= 31), TB positive (n= 43), TB-COVID-19 positive (n= 21) and a set of controls (n= 24). The control group consisted of participants who tested negative for all the above-mentioned diseases screened at the time of the study. Patient recruitment workflow is illustrated as a flowchart in **Figure 1B**. The proportion of males to females was 60% and 40%, respectively. The most frequent symptoms in the COVID-19 group (n=31) were cough (74%), fever (70.9%) and headache (61.3%). The main clinical signs reported during TB-COVID-19 association (n=21) were cough (85.7%), fatigue (80.9%) and headache (76.1%). TB patients (n=43) presented predominant symptoms such as cough (53.5%), fever (46.6%) and chest pain (25.6%). Details of sociodemographic and clinical data are summarized in **Table 1**.

### Serum AST, Urea and D-dimer levels are significantly higher in the TB- COVID-19 association

**Figure 2:**
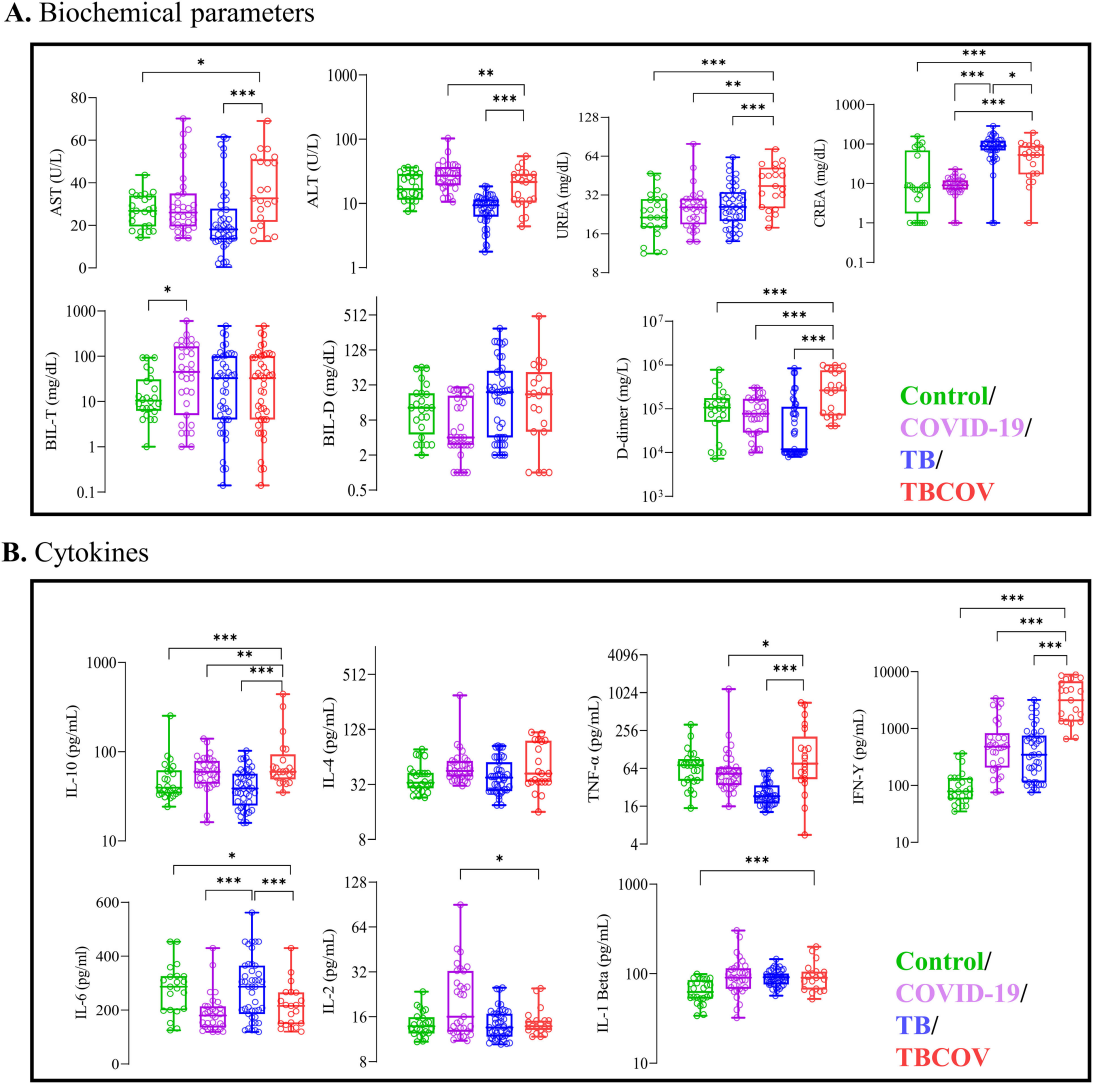
Increased serum levels of AST, UREA, D-dimer and plasma levels of IL-4, IL-10, TNF-α, IFN-γ showed association with Tuberculosis and COVID-19 co-infection. Each individual’s data point is shown, with median values indicated by horizontal lines, the other lines above and below the median represent the interquartile range (IQR) and the minimum and maximum values as appropriate. **(A)** Biochemical parameters such as AST, ALT, D-dimer, Total-bilirubin, Direct-bilirubin, Urea and Crea were measured using spectrophotometry-based assays. **(B)** Plasma levels of anti-inflammatory cytokines are associated with TB/COVID-19association. Sandwich ELISA using Origene kits was employed to measure the systemic levels of anti-inflammatory cytokines (IL-10 and IL-4) and pro-inflammatory (TNF-α, IFN-γ, IL-6, IL-2 and IL-β) in plasma samples of the study population. Unpaired t-test was carried out between various combinations to determine statistically significant differences. AST, Aspartate aminotransferase; ALT, Alanine aminotransferase; CREA, Creatinine; TNF, Tumour necrosis factor; IFN, Interferon; IL, interleukin; TB, tuberculosis; COV, COVID-19; TBCOV, Tuberculosis and Covid-19 association. *p <0.05, **p <0.01, ***p <0.001.

Every study participant’s blood clotting factor and other biochemical parameters were evaluated to determine disease severity in TB and/or COVID-19. To monitor participants’ liver and kidney function, we measured the expression levels of those enzymes (ALT, AST, Urea, creatinine and bilirubin) reflecting these organs’ function (**Figure 2A**). TB-COVID-19 co-infected patients had higher AST levels than TB mono-infected (p<0.001) and controls (p<0.05). AST levels were higher than the normal range (> 34 IU/L) in 50% (10) of participants with TB-COVID-19, 18% (8) with TB and 26% (8) with COVID-19 (**Table 2**). Urea levels in TB-COVID-19 co-infected patients were also significantly higher than in individuals with only TB (p<0.001) or COVID-19 (p<0.01) and controls (p<0.001). It was noticed that all TB-COVID-19 co-infected patients had urea levels exceeding the normal serum concentration (> 20 mg/dL). Conversely, 74% (18) of COVID-19-positive participants and 72% (19) of TB-positive patients exhibited abnormal urea levels (**Table 2**). The COVID-19 group showed significantly elevated ALT levels compared to both the TB (p < 0.0001) and TB-COVID-19(p < 0.001) groups. ALT levels exceeding 36 IU/L were found in 8 COVID-19-positive participants and 2 TB-COVID-19 co-infected participants (**Table 2**). When comparing the AST and ALT profiles across the four study groups, we found that 26% (8 out of 31) of COVID-19-positive participants exhibited AST levels above the normal range. Interestingly, these same individuals also had ALT levels exceeding the normal range. Conversely, in TB-COVID-19 and TB, we found an increased expression level of AST but normal ALT levels in most participants. The D-dimer levels, which are essential for evaluating coagulation abnormalities in clinical settings, were measured among the study participants. In this study, over 53% (18) of TB-positive and 57% (12) TB-COVID-19 co-infected participants demonstrated elevated D-dimer levels (> 0.5 mg/dL) compared to just 25% (6) of control participants. Notably, a small subset of COVID-19 patients also exhibited abnormal D-dimer levels, highlighting the test’s significance in this context. Serum creatinine levels were significantly higher in TB positive patients than those with COVID-19 (p<0.001) and TB-COVID-19 association (p<0.05) (**Figure 2A**). Elevated creatinine levels exceeding 1.4 mg/dL were noted in some of the patients: 16% (5) of those with COVID-19, 14% (6) with TB, and 19% (4) of those co-infected with TB and COVID-19 (**Table 2**). Plasma levels of total bilirubin were significantly higher in COVID-19 mono-infection compared to control (P<0.05), whilst no significant difference was observed between the COVID-19, TB and TB-COVID-19 association groups. Total serum bilirubin levels were abnormal (> 1.2 mg/dL) in three subgroups: 32% (10) COVID-19-positive participants, 23% (10) of TB-positive participants, and 43% (9) of the TB-COVID-19 co-infected group (**Table 2**). Furthermore, direct bilirubin levels were elevated (>0.5 mg/dL) in 32% (14) of TB-positive participants and 24% (5) of TB-COVID-19 patients, in contrast to none of COVID-19 patients and 21% (5) of controls (**Table 2**). These results indicate liver and kidney function damage in TB-COVID-19 co-infected patients.

**Table 2:** Comparison of liver, kidney, and coagulation test ranges across study groups: Control, COVID-19, TB, and TB-COVID-19.

		Control (n=24)		COVID-19 (n=31)		TB (n=43)		TB-COVID-19 (n=21)	
Biochemical parameters	Normal range	Within normal range n (%)	Above normal range n (%)	Within normal range n (%)	Above normal range n (%)	Within normal range n (%)	Above normal range n (%)	Within normal range n (%)	Above normal range n (%)
ALT	7-50 IU/L	24 (100)	0 (0)	23 (74)	8 (26)	43 (100)	0 (0)	19 (90)	2 (10)
AST	5-40 IU/L	23 (96)	1 (4)	23 (74)	8 (26)	35 (81)	8 (19)	11 (52)	10 (48)
Urea	5-20 mg/dL	10 (42)	14 (58)	8 (26)	23 (74)	12 (28)	31 (72)	1 (5)	20 (95)
Creatinine	0.7-1.3 mg/dL	23 (96)	1 (4)	26 (84)	5 (16)	37 (86)	6 (14)	17 (80)	4 (20)
Direct Bilirubin	<0.5 mg/dL	19 (79)	5 (21)	31 (100)	0 (0)	29 (67)	14 (33)	16 (76)	5 (24)
Total bilirubin	<1 mg/dL	23 (96)	1 (4)	21 (68)	10 (32)	33 (77)	10 (23)	12 (57)	9 (43)
D-dimer	0.2-0.5 mg/dL	20 (83)	4 (17)	30 (97)	1 (3)	20 (47)	23 (53)	9 (43)	12 (57)

This table displays the biochemical parameters for liver function, kidney function, and coagulation profiles for each participant, categorized into Control, COVID-19, TB, and TB-COVID-19 groups. The values are compared against established reference ranges from the literature, highlighting deviations and potential abnormalities associated with each condition.

### Higher plasma IL10, IFN-γ and TNF-α levels are associated with *Mycobacterium tuberculosis* and SARS-CoV-2 co-infections

Given the fact that disease severity can be determined by both tissue or organ damage and an exacerbated immune response, this study evaluated immune response involved during TB and/or COVID-19. To monitor the inflammation score, plasma IFN-γ, TNF-α, IL-6, IL-10, IL-4, IL-2 and IL-1β levels were observed between study groups. Plasma levels of two pro-inflammatory cytokines (TNF-α and IFN-γ) were found to be significantly higher in the TB-COVID-19 co-infected group when compared to the mono-infected groups; COVID-19 (p<0.05 and p<0.001) and TB (p<0.001 and p<0.001) positive groups respectively. In the COVID-19 group, plasma IL-2 levels were significantly higher than TB-COVID-19 (p<0.05) co-infected participants. Plasma IL-1β levels did not vary across the COVID-19, TB and TB-COVID-19 groups but were significantly lower in control participants (p<0.001) compared to TB-COVID-19 co-infected participants. TB-positive participants expressed significantly higher plasma IL-6 levels compared to TB-COVID-19 (p<0.001) and COVID-19 (p<0.001) positive participants. Among the two anti-inflammatory cytokines that were measured in this study, plasma IL-10 levels were significantly higher in TB-COVID-19 co-infected patients compared to the TB (p<0.0001) and COVID-19 (p<0.01) mono-infected patients. Plasma IL-4 levels expressed by COVID-19, TB and TB-COVID-19 patients did not vary significantly (**Figure 2B**). The above results indicate an imbalance in immune response during Mtb and SRAS-CoV-2 co-infection.

### Wild-type, heterozygous, and double-mutant genotypes in 07 SNPs (rs2285666, rs6632677, rs4646116, rs4646140, rs147311723, rs2074192 and rs4646142) in the *ace2* gene showed significant variations in distribution across the study groups

**Figure 3:**
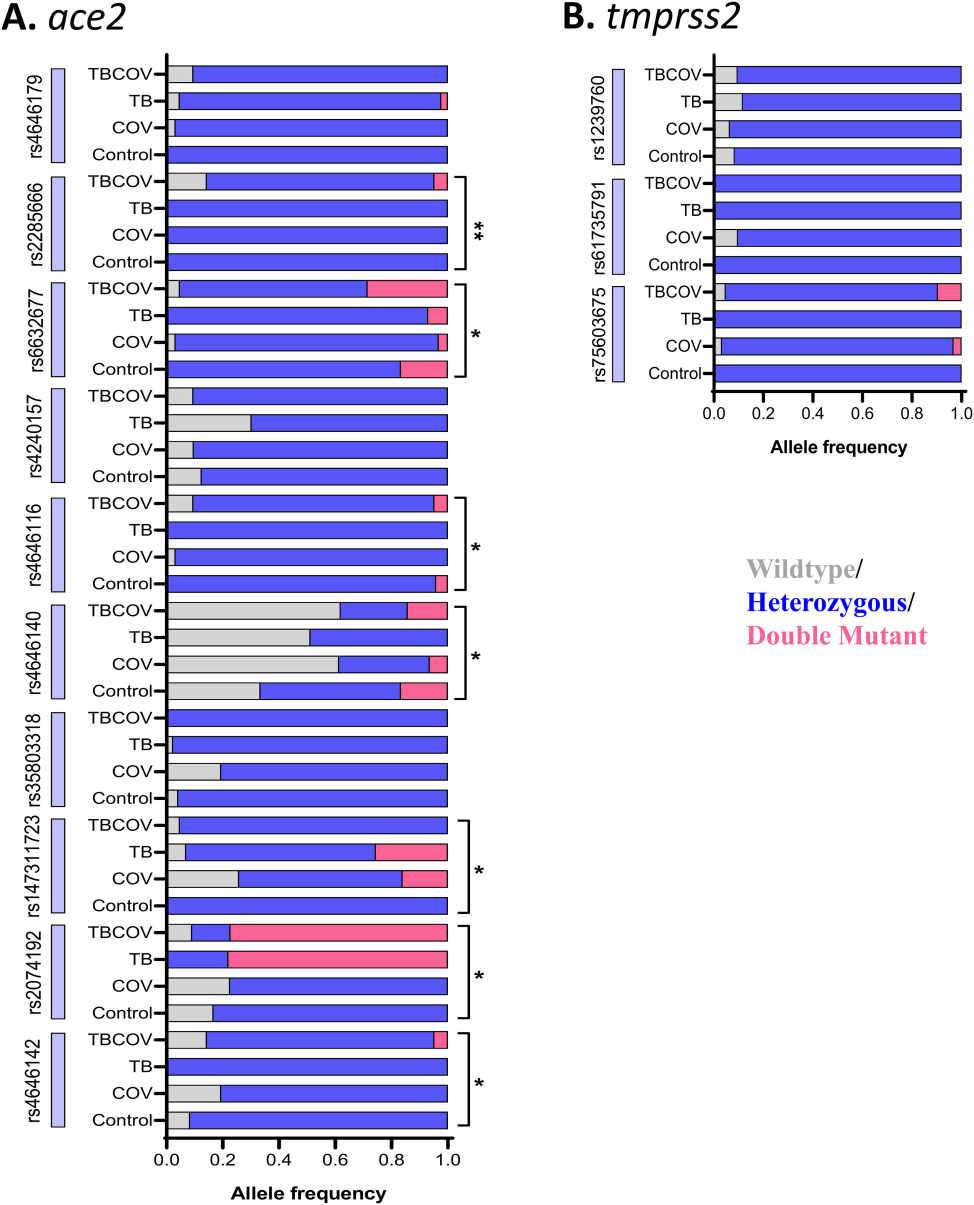
Wild-type, heterozygous, and double-mutant genotypes in seven SNPs (rs2285666, rs6632677, rs4646116, rs4646140, rs147311723, rs2074192 and rs4646142) in ace2 gene showed significant variations in distribution across the four study groups: Genomic DNA isolated using Zymo Human DNA Isolation Kit was subjected to quantitative real-time PCR with TaqMan probes. TaqMan SNP Genotyping Assays were employed to identify SNPs in *ace2* and *tmprss2* genes. **(A)** specific genotype frequencies in *ace2.* **(B)** specific genotype frequencies in *tmprss2*. Multigroup analysis was carried out using the Chi-square test, and SNPs having a p-value of less than 0.05 were considered to have a significantly perturbed presence between the groups (Control, TB, COV, and TBCOV). TB, Tuberculosis positive; COV, COVID-19 positive and TBCOV, Tuberculosis and COVID-19 association. Asterisks represent p-values determined after multivariate analysis that was done using the Chi-square test comparing the mutation patterns in the four groups, namely, control, TB, COVID-19, and TB-COVID-19.

To identify the common mutations in *ace2* and *tmprss2* genes present within a cohort of the Cameroonian population, we identified frequent SNPs of these genes which have been reported to be associated to COVID-19 susceptibility and/or severity. The mutations in allelic expression of 10 SNPs positions in *ace2* (rs2285666, rs4240157, rs4646142, rs4646116, rs6632677, rs4646140, rs147311723, rs2074192, rs35803318, rs4646179) and 03 in *tmprss2* genes (rs12329760, rs75603675, rs61735791) were profiled in every study participant (**Figures 3A, B**). The principal findings of our study, detailed in **Supplementary Table 1**, revealed a significant statistical association among the four groups concerning genotypes for seven SNPs, including rs6632677 (p<0.05), rs4646116 (p<0.05), rs4646142 (p< 0.05), rs2074192 (p< 0.05), rs147311723 (p< 0.05), rs4646140 (p< 0.05), and rs2285666 (p=0.004). The double mutant alleles of the *ace2* gene, specifically rs4646140 (GG) and rs6632677 (GG), were more prevalent in the TB-COVID-19 than in COVID-19 group (**Figure 3**).

### Association between the seven (rs2285666, rs6632677, rs4646116, rs4646140, rs147311723, rs2074192 and rs4646142) *ace2* gene SNPs, which exhibited variations across the study groups, and cytokine expression levels in TB-COVID-19 individuals

**Figure 4:**
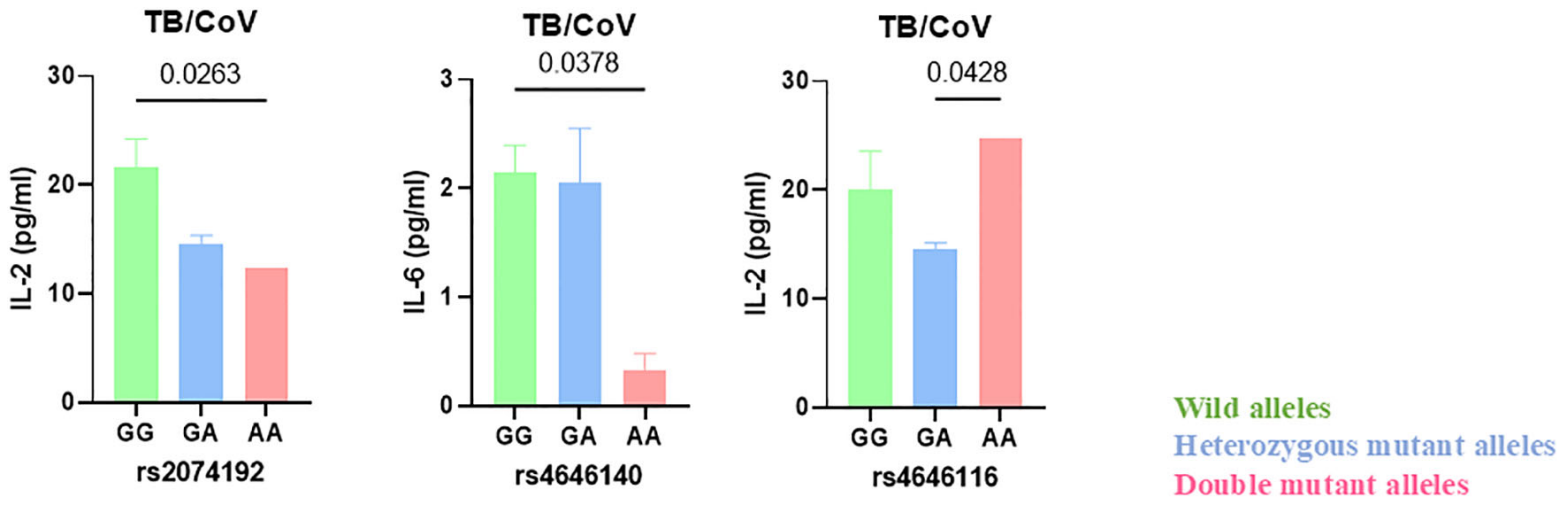
Reduced IL-6 and IL-2 levels linked to double mutant alleles of ACE2 gene polymorphisms rs4646140 and rs2074192 in TB-COVID-19 co-infected patients. The wild-type, heterozygous, and double-mutant genotypes in seven SNPs (rs2285666, rs6632677, rs4646116, rs4646140, rs147311723, rs2074192 and rs4646142) in ace2 gene were associated with expression levels of some pro-inflammatory (IL-6, IL-2, TNF-α and IFN-γ) and anti-inflammatory (IL-10) cytokines in individuals with both TB and COVID-19. Only statistically significant association are reported. The letters on the x-axis denote specific genotypes (wild-type, heterozygous mutant and double mutant). The bars represent the median values of cytokine levels, with error bars indicating the interquartile range (IQR). Horizontal lines and corresponding values above the bars indicate statistical comparisons, with p-values provided to highlight significant differences between groups.

This study evaluated whether the wild-type, heterozygous, and double-mutant genotypes in these SNPs in *ace2* gene impact variations in the expression levels of some pro-inflammatory (IL-6, IL-2, TNF-α and IFN-γ) and anti-inflammatory (IL-10) cytokines in individuals with both TB and COVID-19. Only statistically significant association were reported in **Figure 4**. Our most significant findings include the association of double mutant alleles of rs4646140 and rs2074192 in the ace2 gene with decreased IL-6 and IL-2 expression levels respectively in TB-COVID-19 participants. Additionally, the double mutant alleles of rs4646116 were linked to elevated IL-2 levels (**Figure 4**).

## Discussion

Given the fact that TB and COVID-19 share similar signs and symptoms, misdiagnosis of disease due to clinical parameters alone is likely to be prevalent. All participants from the diseased group in this study had a high frequency of cough, fever, headache and fatigue, consistent with earlier reports (20–22). It has been documented that kidney and liver function could be altered during COVID-19 and TB infections through increased AST, ALT, urea, creatinine and bilirubin levels. In this study, increased serum AST, urea and D-dimer levels were associated with Mtb and SARS-CoV-2 co-infection. Significantly higher serum AST levels observed in the TB-COVID-19 patients in this study corroborated earlier reports (23–25). Higher AST levels in disease groups could be associated with worse outcomes (18). Higher serum urea levels in TB-COVID-19 co-infected patients in this study corroborated a previous report (26). Increased D-dimer levels in patients with concurrent TB and COVID-19 in this study may indicate elevated thrombotic risks. This is consistent with findings reported in Pakistani populations (27). The elevation of D-dimer, a fibrin degradation product, often correlates with inflammation and coagulopathy, critical considerations in managing patients with these comorbidities (28). An increase in expression levels of D-dimer reported in this study among TB-COVID-19 co-infected patients is generated and reported in severe inflammatory responses involving other diseases. Monitoring D-dimer levels could be essential for assessing thrombotic risk and guiding treatment strategies. As observed in various studies, elevated serum ALT levels in COVID-19 patients can suggest underlying liver dysfunction (23, 28, 29). COVID-19 has been associated with hepatic impairment, and increased ALT is often a marker of liver injury. Zhang et al. noted similar findings, highlighting the importance of monitoring liver function in COVID-19 patients (30).

One significant finding reported in this study was an imbalance immune response in TB-COVID-19 co-infected patients demonstrated by high levels of IFN- γ, TNF-α and IL-10. IFN-γ’s role is partially reflected by the fact that it increases the production of proinflammatory cytokines via activation of the JAK-STAT pathway, leading to clinical manifestations of disease (31). Elevated IFN-γ levels in TB-COVID-19 may contribute to the abnormal systemic inflammatory responses that increase disease severity. While IFN-γ production shows subject-specific variations, reduced levels were reported in active TB patients (30, 32). Also, TNF-α, which is a key cytokine involved in inflammatory responses in TB and COVID-19, demonstrated higher levels in TB-COVID-19 positive patients. A recent study in mice models found that combining TNF-α and IFN-γ induced a cytokine-mediated inflammatory cell death signaling pathway via JAK-STAT1 (19). Increased TNF-α production could facilitate viral infection and exacerbate organ damage. Some studies suggest that blocking TNF-α with inhibitors can strongly modulate the balance between effector T cells and regulatory T cells, potentially enhancing immune regulation. In addition, inhibiting TNF-α has been shown to reduce levels of IL-6, IL-1, adhesion molecules, and vascular endothelial growth factor (VEGF) in rheumatoid arthritis (RA) patients, as reported in various clinical cohort studies on COVID-19 (31).Recent research has demonstrated that the synergy between TNF-α and IFN-γ is crucial for triggering robust cell death by activating the JAK/STAT1/IRF1 axis in human monocytic cells (THP-1) and primary human umbilical vein endothelial cells (19). Other pro-inflammatory cytokines such as IL-6, IL-2 and IL-1β showed varying expression levels among the diseased participants. Higher plasma IL-6 levels observed in TB patients discriminated efficiently from the other study participants (33–35). IL-6 levels associated with pro-inflammatory cytokine variants contribute to the cytokine storm, deteriorating COVID-19 outcomes (35, 36). The higher levels of IL-2 expressed during COVID-19 in this study corroborate other studies (37–40). A previous report demonstrated that IL-2 levels helped determine the prognosis of lung damage in influenza A patients (41). IL-1β, another pro-inflammatory cytokine, was expressed at higher levels in the TB, COVID-19 and TB-COVID-19 co-infection groups compared to the controls. This finding aligns with previous research on TB and COVID-19 patients (42, 43). IL-10, an anti-inflammatory cytokine, was found at significantly higher levels in TB-COVID-19 patients than other groups. Previous studies have also reported elevated IL-10 levels in TB-COVID-19 patients compared to those with either TB or COVID-19 alone, regardless of severity (27, 28). Plasma IL-4 levels in TB-COVID-19 co-infected patients were significantly higher than in the other diseased and control groups, corroborating earlier reports (44).

Despite data regarding variations in genotype frequencies of *ace2* and *tmprss2* during SARS-CoV-2 infection, limited information is available about their implication in TB and COVID-19 association. Our data adds considerable insight to the literature on mutations of *ace2* and *tmprss2* involved in TB- COVID-19 association pathogenesis. The present study sought to identify genotypic variations in human *ace2* and *tmprss2* genes, associate them with immune response and correlate these with susceptibility to Mtb and SARS-CoV-2 co-infection. Numerous studies and reports have consistently demonstrated that the host Ace2 receptor plays a pivotal role as the primary entry point for the SARS-CoV-2 virus. At the same time, Tmprss2 has been identified as a crucial enzyme responsible for facilitating the activation of the viral spike protein, enabling its fusion with the host cell membrane and subsequent viral entry (10, 25, 45). Specific changes in the *ace2* and *tmprss2* gene sequences, which may increase the binding affinity and/or expression levels, may affect the entry of the SARS-CoV-2 (46). Populations with specific SNPs in the *ace2* and *tmprss2* genes have shown increased susceptibility to COVID-19 and TB-COVID-19 association (47–49). Reports by Chen and collaborators in the evaluation of the relationship between genetic variants of the *ace2* gene and circulating levels of Ace2 found several allele frequencies of *ace2* coding across different populations (South Asian, East Asian, African, European, and mixed American populations). The allele frequencies of 11 of the 15 eQTLs (expression quantitative trait locus) that were linked to expression were more significant in East Asians (0.73–0.99) than in Europeans (0.44–0.65), which is indicative of the differential susceptibility to SARS-CoV-2 among different cultures (50). Such reports on the structural and regulatory variants of *ace2* and *tmprss2* conferring susceptibility to COVID-19 from the Cameroonian population are limited. Reports showed that African populations are genetically predisposed to low *ace2* and *tmprss2* expression, partly explaining the lower incidence of COVID-19 (18). On the other hand, allelic frequencies contributing to higher *ace2* and *tmprss2* expressions in South Asian, Southeast Asian, and East Asian populations reported higher infection rates (51, 52). In this study, we monitored polymorphism patterns in *ace2* and *tmprss2* to find a correlation with higher genetic susceptibility to TB-COVID-19. The high incidence rate of COVID-19 and TB-COVID-19 co-infected males could be potentially attributed to the presence of the *ace2* gene on the X-chromosome (53). A recent report demonstrated an inverse correlation between *ace2* expression levels and estrogen levels in SARS-CoV-2 patients (54). Estrogen may contribute to the suppression of *ace2* expression thus explaining and might partly explain the protective factors in females against COVID-19 (55). We observed the association of double mutant alleles of rs4646140 and rs2074192 in the ace2 gene with decreased IL-6 and IL-2 expression levels respectively in TB-COVID-19 participants. This may confer a protective effect against severe COVID-19 or worse outcome during these co-infections. Also, the double mutant alleles (AA) of rs4646116 were responsible for increased expression level of IL-2 in TB-COVID-19 patients. While IL-2 is crucial for T cell activation and expansion, persistently high levels can lead to T cell exhaustion, reducing their ability to effectively combat the virus hence prolonging infection. High IL-2 levels have been associated with severe COVID-19 cases, suggesting it could serve as a biomarker for disease severity (41). Monitoring IL-2 levels might help identify patients at risk of progressing to severe illness. The double mutant alleles (AA) of SNP rs2074192 in *ace2* were common in TB-COVID-19 co-infected patients. This implies that the AA allele may play a role in the susceptibility or pathophysiology of co-infection with these two diseases hence suggesting a potential genetic predisposition that could influence how these individuals respond to these infections. However, further research would be needed to fully understand the biological mechanisms and implications behind this finding.

It is essential to acknowledge the limitations of a small sample size, and the fact that participants were not monitored over an extended period as it can restrict the generalizability of the findings. The lack of long-term monitoring limits the ability to observe potential changes in biomarkers or disease progression over time. Future research with larger, longitudinal cohorts would be valuable to confirm and expand on the relationships between these SNPs and other research indices. Also, the SNP distribution evaluated in this study may be population-specific and may differ among different populations. The survey of polymorphisms from diverse genetic backgrounds might explain the vulnerability to diseases. This study has a limited scope of cytokine profiling due to the restricted number of cytokines analyzed. While several key cytokines were investigated, the study did not include a comprehensive panel of cytokines that could offer a more complete picture of the immune response. Furthermore, the cytokine levels measured were specific to certain time points, and longitudinal investigation could have provided more insights into cytokine dynamics during disease progression or treatment.

In summary, this study indicates that the double mutant alleles of rs2074192 and rs4646140 in the *ace2* gene reduced IL-2 and IL-6 production in TB-COVID-19 individuals which could potentially lead to milder disease outcomes in these individuals. Whilst the double mutant alleles of rs4646116 increased inflammatory response through increase IL-2 production which may lead to deleterious outcomes in TB-COVID-19 individuals with time. These findings highlight the need for a human genetics initiative to understand better the genetic factors influencing susceptibility and/or severity during TB-COVID-19 association. This could inform prevention and treatment strategies during the future pandemic.

## Data Availability

All relevant data are within the manuscript and its **Supplementary Information files** that were uploaded during the review process.
