# Supplementary material for: Mutations in ace2 gene modulate cytokine levels and alter immune responses in Mycobacterium tuberculosis and SARS-CoV-2 co-infection: a Cameroonian cohort
Source: Front Immunol. 2025 Mar 24;16:1533213. doi: 10.3389/fimmu.2025.1533213 (PMC11973369; doi:10.3389/fimmu.2025.1533213)
Supplement: Supplementary file 1 [file Table1.docx]

**Mutations in ace2 gene Modulate Cytokine Levels and Alter Immune Responses in Mycobacterium tuberculosis and SARS-CoV-2 co-infection: A Cameroonian cohort.**

**Authors:** Mary Ngongang Kameni^1,2,3^, Eric Berenger Tchoupe^1,4^, Severin Donald Kamdem^1,5^, Nikhil Bhalla^3^, Jean Paul Assam Assam^2,^ Arnaud Njiguet Tepa^1^, Fuh Roger Neba^1^, Ranjan Kumar Nanda^3^, Anthony Afum-Adjei Awuah^6,7,8^, John Amuasi^6,7,8^, Palmer Masumbe Netongo^1,9,10*^

**Supplementary Document.**

**Supplementary Table 1:** Genotype frequencies distribution of single nucleotide polymorphisms of *ace2* and *tmprss2* genes. Chi-square test was used to determine statistically significant differences in genotype frequencies among the groups (Control, COV, TB, and TBCOV). A *p-value* of less than 0.05 was considered significant.

|  | | **Groups** | **Genotypes** | | | **P-value** |
| --- | --- | --- | --- | --- | --- | --- |
|  |  |  | **Homozygous GG n (%)** | **Heterozygous AG n (%)** | **Homozygous AA n (%)** |  |
| ***ace2*** | **rs4646142** | Control (n= 24) | 2 (8.4) | 22 (91.6) | 0 (0) | <0.05 |
|  |  | COV (n = 31) | 6 (19.4) | 25 (80.6) | 0 (0) |  |
|  |  | TB (n = 43) | 0 (0) | 43 (100) | 0 (0) |  |
|  |  | TBCOV (n = 21) | 3 (14.3) | 17 (80.9) | 1 (4.8) |  |
|  | **rs2074192** | Control (n= 24) | 4 (16.7) | 20 (83.3) | 0 (0) | <0.05 |
|  |  | COV (n = 31) | 7 (22.5) | 24 (77.4) | 0 (0) |  |
|  |  | TB (n = 43) | 0 (0) | 9 (20.9) | 32 (74.4) |  |
|  |  | TBCOV (n = 21) | 2 (9.5) | 3 (14.3) | 17 (80.9) |  |
|  | **rs147311723** | Control (n= 24) | 0 (0) | 24 (100) | 0 (0) | <0.05 |
|  |  | COV (n = 31) | 8 (25.8) | 18 (58.1) | 5 (16.1) |  |
|  |  | TB (n = 43) | 3 (6.8) | 29 (67.4) | 11 (25.6) |  |
|  |  | TBCOV (n = 21) | 1 (4.8) | 20 (95.2) | 0 (0) |  |
|  | **rs35803318** | Control (n= 24) | 1 (4.2) | 23 (95.8) | 0 (0) | ns |
|  |  | COV (n = 31) | 6 (19.4) | 25 (80.6) | 0 (0) |  |
|  |  | TB (n = 43) | 1 (2.3) | 42 (97.7) | 0 (0) |  |
|  |  | TBCOV (n = 21) | 0 (0) | 21 (100) | 0 (0) |  |
|  | **rs4646140** | Control (n= 24) | 8 (33.3) | 12 (50) | 4 (16.6) | <0.05 |
|  |  | COV (n = 31) | 19 (61.3) | 10 (32.3) | 2 (6.5) |  |
|  |  | TB (n = 43) | 22 (51.2) | 21 (48.8) | 0 (0) |  |
|  |  | TBCOV (n = 21) | 13 (61.9) | 5 (23.8) | 3 (14.2) |  |
|  | **rs4646116** | Control (n= 24) | 0 (0) | 23 (95.8) | 1 (4.2) | <0.05 |
|  |  | COV (n = 31) | 1 (3.2) | 30 (96.8) | 0 (0) |  |
|  |  | TB (n = 43) | 0 (0) | 43 (100) | 0 (0) |  |
|  |  | TBCOV (n = 21) | 2 (9.5) | 18 (85.7) | 1 (4.8) |  |
|  |  |  | Homozygous CC | Heterozygous CG | Homozygous GG |  |
|  | **rs4240157** | Control (n= 24) | 3 (12.5) | 21 (87.5) | 0 (0) | ns |
|  |  | COV (n = 31) | 3 (9.7) | 28 (90.3) | 0 (0) |  |
|  |  | TB (n = 43) | 13 (30.2) | 30 (69.8) | 0 (0) |  |
|  |  | TBCOV (n = 21) | 2 (9.5) | 19 (90.5) | 0 (0) |  |
|  | **rs6632677** | Control (n= 24) | 0 (0) | 20 (83.3) | 4 (16.7) | <0.05 |
|  |  | COV (n = 31) | 1 (3.2) | 29 (93.5) | 1 (3.2) |  |
|  |  | TB (n = 43) | 0 (0) | 40 (93.0) | 3 (6.9) |  |
|  |  | TBCOV (n = 21) | 1 (4.8) | 14 (66.7) | 6 (28.6) |  |
|  |  |  | Homozygous CC | Heterozygous CA | Homozygous GG |  |
|  | **rs2285666** | Control (n= 24) | 0 (0) | 24 (100) | 0 (0) | 0.004 |
|  |  | COV (n = 31) | 0 (0) | 31 (100) | 0 (0) |  |
|  |  | TB (n = 43) | 0 (0) | 43 (100) | 0 (0) |  |
|  |  | TBCOV (n = 21) | 3 (14.3) | 17 (80.9) | 1 (4.8) |  |
|  |  |  | Homozygous AA | Heterozygous AG | Homozygous GG |  |
|  | **rs4646179** | Control (n= 24) | 0 (0) | 24 (100) | 0 (0) | ns |
|  |  | COV (n = 31) | 1 (3.2) | 30 (96.8) | 0 (0) |  |
|  |  | TB (n = 43) | 2 (4.7) | 40 (93) | 1 () |  |
|  |  | TBCOV (n = 21) | 2 (9.5) | 19 (90.5) | 0 (0) |  |
| ***tmprss2*** |  |  | Homozygous CC | Heterozygous CA | Homozygous AA |  |
|  | **rs75603675** | Control (n= 24) | 0 (0) | 24 (100) | 0 (0) | ns |
|  |  | COV (n= 31) | 1 (3.3) | 29 (93.5) | 1 (3.3) |  |
|  |  | TB (n = 43) | 0 (0) | 43 (100) | 0 (0) |  |
|  |  | TBCOV (n= 21) | 1 (4.76) | 18 (85.7) | 2 (9.5) |  |
|  |  |  | Homozygous CC | Heterozygous CA | Homozygous AA |  |
|  | **rs61735791** | Control (n= 24) | 0 (0) | 24 (100) | 0 (0) | ns |
|  |  | COV (n= 31) | 3 (9.8) | 28 (90.3) | 0 (0) |  |
|  |  | TB (n = 43) | 0 (0) | 43 (100) | 0 (0) |  |
|  |  | TBCOV (n= 21) | 0 (0) | 21 (100) | 0 (0) |  |
|  |  |  | Homozygous CC | Heterozygous CT | Homozygous TT |  |
|  | **rs12329760** | Control (n= 24) | 2 (8.3) | 22 (91.7) | 0 (0) | ns |
|  |  | COV (n= 31) | 2 (6.5) | 29 (93.5) | 0 (0) |  |
|  |  | TB (n= 43) | 5 (11.6) | 38 (88.4) | 0 (0) |  |
|  |  | TBCOV (n=21) | 2 (9.5) | 19 (90.5) | 0 (0) |  |

Abbreviations: TB: Tuberculosis positive; COV: COVID-19 positive and TBCOV: Tuberculosis and COVID-19 association.

***End of Supplementary Document***
